# Tracking distinct microglia subpopulations with photoconvertible Dendra2 in vivo

**DOI:** 10.1186/s12974-021-02285-x

**Published:** 2021-10-15

**Authors:** Eric B. Miller, Sarah J. Karlen, Kaitryn E. Ronning, Marie E. Burns

**Affiliations:** 1 Center for Neuroscience, University of California, 1544 Newton Court, Davis, CA 95618 USA; 2 Department of Cell Biology and Human Anatomy, University of California, Davis, CA 95616 USA; 3 Department of Ophthalmology & Vision Science, University of California, Davis, CA 95616 USA

**Keywords:** Retina, Photoreceptor, Microglia, Fluorescent protein, Imaging

## Abstract

**Background:**

The ability to track individual immune cells within the central nervous system has revolutionized our understanding of the roles that microglia and monocytes play in synaptic maintenance, plasticity, and neurodegenerative diseases. However, distinguishing between similar subpopulations of mobile immune cells over time during episodes of neuronal death and tissue remodeling has proven to be challenging.

**Methods:**

We recombineered a photoconvertible fluorescent protein (Dendra2; D2) downstream of the Cx3cr1 promoter commonly used to drive expression of fluorescent markers in microglia and monocytes. Like the popular *Cx3cr1*–*GFP* line (*Cx3cr1*^+*/GFP*^), naïve microglia in *Cx3cr1*–*Dendra2* mice (*Cx3cr1*^+*/D2*^) fluoresce green and can be noninvasively imaged in vivo throughout the CNS. In addition, individual D2-expressing cells can be photoconverted, resulting in red fluorescence, and tracked unambiguously within a field of green non-photoconverted cells for several days in vivo.

**Results:**

Dendra2-expressing retinal microglia were noninvasively photoconverted in both ex vivo and in vivo conditions. Local in vivo D2 photoconversion was sufficiently robust to quantify cell subpopulations by flow cytometry, and the protein was stable enough to survive tissue processing for immunohistochemistry. Simultaneous in vivo fluorescence imaging of Dendra2 and light scattering measurements (Optical Coherence Tomography, OCT) were used to assess responses of individual microglial cells to localized neuronal damage and to identify the infiltration of monocytes from the vasculature in response to large scale neurodegeneration.

**Conclusions:**

The ability to noninvasively and unambiguously track D2-expressing microglia and monocytes in vivo through space and time makes the *Cx3cr1*–*Dendra2* mouse model a powerful new tool for disentangling the roles of distinct immune cell subpopulations in neuroinflammation.

**Supplementary Information:**

The online version contains supplementary material available at 10.1186/s12974-021-02285-x.

## Key points

- New mouse for tracking microglia and all mononuclear phagocytes both ex and in vivo within the CNS over time.
- Dendra2 protein is stable enough to survive tissue processing for immunohistochemistry and flow cytometry quantification.
- Simultaneous fluorescence imaging of Dendra2 and light scattering measurements can be used to assess the immune response to retinal damage.
- Chronic in vivo imaging reveals mixed populations of microglia and monocytes during retinal degeneration.

**Supplementary Information:**

The online version contains supplementary material available at 10.1186/s12974-021-02285-x.

## Background

Microglia are the long-lived, yolk-sac derived resident immune cells of the central nervous system (CNS) that prune developing circuits, maintain healthy synaptic contacts in adulthood, and phagocytose debris and pathogens [1–3]. Recent work suggests the existence of microglial subpopulations, which may have important consequences for their baseline function and their response to disease [4–7]. In addition, damage or disease can cause the recruitment of bone-marrow derived monocytes across the blood–retinal barrier, and these monocytes can assume morphologies and expression patterns that are similar to the native microglia yet have distinct cellular functions or susceptibilities to subsequent insults [1, 8–11]. Work in the last 5 years has sought to identify specific molecular markers for subpopulations of microglia, monocytes, and monocyte-derived macrophages, though such markers may be highly dependent on the cells’ activation states and surrounding environments, making their use across models and CNS niches difficult [12, 13].

Dendra2 (D2) is a monomeric, photoconvertible fluorescent protein originally cloned from the soft coral *Dendronephthya* sp. [14–16] with a similar overall structure to that of GFP [17]. Like GFP, the unconverted form of D2 has a peak excitation at 490 nm and a peak emission at 507 nm; however, in D2, short wavelength light induces a structural photoconversion that shifts the spectral properties to longer wavelengths, with a peak excitation of 553 nm and a peak emission of 573 nm [14, 18]. Unlike many other photoswitchable proteins, the conversion from green (gD2) to red (rD2) is permanent, with the red signal only decaying due to protein turnover [16]. Thus, D2 fluorescence acts as a stable, long-lived marker that allows cells to be noninvasively tagged and tracked over space and time.

While D2 has been incorporated into several plant models [19] and used in cell culture work [20], its utilization in living animals has been limited. Examples of in vivo applications include neutrophil trafficking during inflammation in zebrafish [21], quantifying protein turnover rates in vivo in *C. elegans* [22], imaging vascular endothelial cell mitochondria in the mouse brain [23], and tagging leukocytes to track lymph node-homing in mice using fluorescence activated cell sorting (FACS) [24]. The diversity of D2 applications across animal systems underscores the utility of this small, photoconvertible protein for cell identification and discrimination using current fluorescent imaging technology.

To help track individual microglia and monocyte-derived cells over space and time, we developed a mouse line expressing D2 under the Cx3cr1 promoter, the expression of which is commonly used for myeloid lineage tracing both ex vivo and in vivo [25, 26]. In vivo high-resolution retinal imaging of Cx3cr1-driven GFP expression allows individual microglia to be visualized at single cell resolution, but unambiguously following specific microglia over days has not been possible owing to the highly mobile nature of the cells, even in healthy tissue. Here we show that photoconversion of Cx3cr1-driven D2 expression can be used to label specific subpopulations of microglia and monocytes over several days in ex vivo and in vivo retinal imaging applications and flow cytometry assays, allowing us to track and quantify the cellular behavior of these subpopulations across the retina over time.

## Methods

### Animals

All animal procedures were performed in accordance with the University of California, Davis and Biocytogen’s Institutional Animal Care and Use Committees and all relevant guidelines and regulations. *Cx3cr1*–*GFP* (*Cx3cr1*^*GFP/GFP*^) mice were obtained from Jackson Labs (strain #005582), crossed to *C57Bl/6 J* mice (Jackson Labs strain #000664), and genotyped to ensure they did not contain either the rd8 or rd1 mutations. *Cx3cr1*–*Dendra2* mice (*Cx3cr1*^*D2/D2*^) were generated as described below. Both strains were housed under 12/12 h diurnal cyclic light, with food and water available ad libitum. *Arr1*^*−/−*^* Cx3cr1*^+*/D2*^ mice were created by crossing *Arr1*^*−/−*^ mice [27, 28] with *Cx3cr1*^*D2/D2*^ mice. Mice with the Arrestin-1 background were born and maintained in constant darkness before exposure to uniform white light of 200 lx, which initiated retinal degeneration. Both male and female adult animals were used in these studies.

### Generation of the Cx3cr1–Dendra2 mutant mouse

Dendra2 knock-in mice were generated by Biocytogen, Inc. using CRISPR/Cas9 methodology. In brief, to generate the gene targeting vector, single guide RNAs were designed to target two sites of exon 2 of the *Cx3cr1* gene. For each targeting site, candidate sgRNAs were designed by the CRISPR design tool (http://crispr.mit.edu), and screened for on-target activity using UCA™ (Universal CRISPR Activity Assay), a sgRNA activity detection system developed by Biocytogen that is simpler and more sensitive than MSDase assay. The Cas9 mRNA and sgRNAs were transcribed by T7 RNA polymerase by PCR amplification, gel purified, and used as the template for in vitro transcription using the MEGAshortscript T7 kit (Life Technologies) according to the kit protocol. The Cas9 mRNA and sgRNAs were purified using the MEGAclear kit and eluted with RNase-free water. To minimize random integrations, a circular donor vector was employed. The gene targeting vector containing Dendra2–SV40-pA and 2 homology arms of left (1500 bp) and right (1500 bp) each was used as a template to repair the DSBs generated by Cas9/sgRNA. The region coding first 130 amino acids was replaced by Dendra2–SV40-pA.

C57BL/6 N female mice and KM mouse strains were used as embryo donors and pseudopregnant foster mothers, respectively. Super-ovulated female C57BL/6 N mice (3–4 weeks) were mated to C57BL/6 N stud males, and fertilized embryos were collected from the ampullae of the dams. Optimized concentrations of Cas9 mRNA, sgRNAs, and donor vector were mixed and co-injected into the cytoplasm of one-cell stage fertilized eggs. After injection, surviving zygotes were transferred into oviducts of KM albino pseudopregnant females.

Genomic DNA extracted from tails of the offspring was digested with AseI or ScaI (NEB), separated on a 1% agarose gel, and transferred to a positively charged nylon membrane (Hybond N + ; Amersham International plc) for Southern Blot analysis. The blot was hybridized overnight using a DIG Easy Hyb Granules (Roche Applied Science Inc.) at 42 °C with a PCR-generated probe homologous to either the external or internal insertion sequences. Both 3’-external and internal DIG-labeled probes were prepared by PCR using Taq DNA polymerase incorporating DIG-11-dUTP (PCR DIG probe synthesis kit; Roche Applied Science Inc.) according to the manufacturer’s instruction. Hybridization signals were then detected using the DIG Luminescent Detection Kit (Roche Applied Science Inc.). The following labeled primers were used to amplify the 3’-external (422 bp) probe: ACCCAGTTGGCCATGTCCCTT (forward primer) and GGCCTGGGTCTTGCCCTGAC (reverse primer). For the internal (530 bp) probe, the following primers were used: CAGACCGCCAACCTGACCGT (forward primer) and ACGGCGTGCTCGTACAGCTT (reverse primer). Mice bearing successful knock-ins were outcrossed to C57Bl/6 J mice (Jackson Labs) for several generations and genotyped to ensure they did not contain either the rd8 or rd1 mutations commonly found in inbred strains.

### Immunohistochemistry

Mice were sacrificed by carbon dioxide euthanasia and eyes enucleated and submerged in 4% paraformaldehyde at room temperature. After 5 min of fixation, the cornea and lens were removed, and the eyecups were fixed for an additional 20–25 min. Retinas were removed from fixed eyecups and relieving cuts were made for flattening. Retinas were incubated in 1% Triton X-100 in 1X phosphate buffered saline (PBS) overnight at 4 °C, and then blocked with normal serum for 2 h at 37 °C. Retinas were incubated with primary antibodies overnight at 4 °C, washed 3 times in 1X phosphate buffered saline, 0.5% bovine serum albumin, with 0.5% Triton X-100 (PBT) for 15–30 min at room temperature, and incubated in secondary antibody/pre-conjugated primary solution for 1.5–2 h at 37 °C before 3 PBT washes and mounting with ProLong Diamond Antifade with DAPI (Invitrogen). Tissue was stained for GFP (1:300, Rockland) and Iba1 (1:1000, Wako), followed by Alexa Fluor-conjugated secondary antibody (1:300; Invitrogen). Iba1 is a calcium-binding adapter protein used as a standard molecular marker used to identify microglia [29] and GFP was used to locate Dendra2, which is cross-reactive due to their similar structure [17]. Tissue was imaged using a Nikon A1 confocal microscope.

### Ex vivo imaging and photoconversion

Mice were euthanized by carbon dioxide narcosis and the eyes removed. Retinas were excised, cut into a clover-leaf shape for flattening, and placed into glass-bottomed Petri dish filled with Dulbecco's Modified Eagle Medium (DMEM, Sigma Aldrich) supplemented with 10% Fetal Bovine Serum (FBS; Corning) and secured with overlying mesh. The dish was then placed in an imaging chamber (Thermo Scientific) with 10% CO_2_ and maintained at 37 °C. Retinas were imaged on a Nikon A1 imaging system for up to 5 h. Dendra2 was converted at the lowest 405 nm laser power setting, which corresponded to 3 µW at the imaging stage for 2 min. Converted area sizes ranged from cell soma/branches (~ 20 × 20 µm) to multiple cells (~ 320 × 320 µm). Excitation and conversion lasers were simultaneously used for some spectral imaging experiments to measure the rate of Dendra2 conversion.

### Flow cytometry

Flow cytometry was performed using a protocol modified from an established method [8, 30]. After dissection, each retina was incubated in 1 mL of digestion buffer containing: Hank’s Balanced Salt Solution (10–547F, Lonza), 5% FBS, 10 mM HEPES, 0.7 mg/mL calcium chloride, 1.5 mg/mL Collagenase A (10103586001, Roche), and 0.1 mg/mL DNase I (10104159001, Roche) at 37 °C for 15 min. Following incubation, each retina was gently dissociated, and the resulting single-cell suspension was washed, filtered through a 70 µm cell strainer, centrifuged at 350×*g* for 5 min, and resuspended in PBS. Cells were stained for viability (Zombie Viability NIR, 423105, Biolegend), and blocked with Fc light chain antibodies (14016186, eBiosciences), supplemented with normal rat serum and normal mouse serum. Cells were then incubated with Brilliant Violet 421 anti-mouse CD45 (103133, Biolegend) and Alexa Fluor 647 anti-mouse/human CD11b (101220, BioLegend). Cell suspensions were washed in PBS containing 0.5% Bovine Serum Albumin (BSA), centrifuged at 350×*g* for 5 min, and resuspended in 0.5% BSA in PBS with 1:50 EDTA. Bead controls were created using AbC Total Antibody Compensation Bead Kit (A10497, Invitrogen) and ArC Amine Reactive Compensation Bead Kit (A10628, Invitrogen). Data were acquired on a Cytoflex flow cytometer and analyzed with FlowJo software (Tree Star). Each retina was considered a single sample; 200,000 events were analyzed for each sample.

### In Vivo imaging and Dendra2 conversion

A custom-built, combined SLO and OCT system was used for fluorescence retinal imaging and Dendra2 conversion equipped with an OBIS LX/LS Laser Box (Coherent Inc. Santa Clara, CA, USA) with 3 laser diode modules [31]. A 488 nm laser diode module (120 µW at the mouse pupil) was used to excite green Dendra2, and a 561 nm laser diode module was used to excite red Dendra2. The 561 nm beam power varied between 130 and 200 µW at the mouse pupil. A 405 nm laser diode module was used to illuminate the retina and convert Dendra2 from green to red. The power of the 405 nm exposure ranged from 20 to 100 µW for 5–10 min, and the converted area ranged from 0.13 mm^2^ to 1.2 mm^2^. Consequently, the light fluence varied from 7.5 to 22.5 J/cm^2^ at the cornea, resulting in 3–9 J/cm^2^ radiant exposure at the retinal surface when corrected for cornea and lens transmittance [32].

For imaging, the mice were anesthetized with 2–2.5% isoflurane and positioned on a heating pad at 37 °C. The eyes were dilated and cyclopledged with tropicamide and phenylephrine and corneal hydration was maintained with Hypromellose gel (GenTeal Tears Severe; Alcon). Widefield SLO and OCT images spanned 51° of visual angle (2193 µm), while digitally “zoomed” images ranged between 200 µm for obtaining spectra or 728 µm for imaging the injury and response fields in the light damage model.

A superluminescent diode (860 nm, 132 nm bandwidth; Broadlighter T-860HP; Superlum) was used for OCT imaging (600 µW at pupil) and causing damage (8mW at pupil) as described previously [33]. OCT images were then processed and flattened for display [33, 34]. En face OCT images were taken from the outer nuclear layer, while B-scans were taken from the same location for each timepoint in both models. For the near infrared (NIR) light damage model, B-scans were taken through the center of the injury field.

Spectral imaging was performed with an OceanOptics Spectrometer (QE65000 OceanOptics Inc. Dunedin, FL, USA) over a small field of view (i.e., hyperspectral images were not created as they were in ex vivo spectra). The 488 nm and 561 nm excitation sources were used separately with set of a dichroic mirrors Di01–R488/561 (Semrock, Rochester, NY), with a 503 LP emission filter for the 488 nm excitation and a 561 nm LP emission filter for the 561 nm excitation (Semrock, Rochester, NY). Conversion could not occur during spectral imaging, because different fibers were required for 405 vs 488/561 nm source delivery. For each data set, 20 spectra with 1000 ms integration time were obtained and averaged.

### Fluorescence image analysis

Images from both ex vivo and in vivo experiments were analyzed with ImageJ (FIJI; NIH; [35]) and Python (SciPy stack and Sci-Kit Image; [36]). Ex vivo images taken on the Nikon A1 microscope were exported to tiff format using NIS elements viewer (Nikon Instruments, Inc.). For in vivo images, backgrounds were adjusted to provide similar overall contrast. To measure the SLO fluorescence signal in the injury and response field, an ROI 728 × 728 µm, centered on the center of the injury field in the widefield images, was created. The background was determined using the Rolling Ball background subtraction function was created using a radius of 50px and averaged to produce the background value. The pixel intensity was then averaged in the ROI and divided by the background value to produce the signal strength. This normalization served to account for differences in excitation laser power or other issues that created changes in raw signal intensity.

### Spectral image analysis

Spectral images were analyzed with ImageJ and Python. For hyperspectral ex vivo image and movie display, the NIS elements viewer was used to select the channels with the peak gD2 and rD2 fluorescence, then exported to tiffs and appropriate Look-Up Tables (LUTs) were applied. For analysis, the full hyperspectral image was exported as tiff and analyzed with custom-written Python code. For time series of D2 conversion, the spectra in 10 nm width bins are display for each scan. Spectra were color-coded from green to red based on scan number and each scan took ~ 1 s. Twenty scans were taken prior to 405 nm exposure and were not displayed. To determine the time course of gD2 loss and rD2 gain, the 10 nm-wide bins that included the peak gD2 and rD2 emission wavelengths were taken and displayed, including the 20 pre-405 nm exposure scans.

To quantify converted rD2, spectral data were unmixed using the theoretical rD2 and autofluorescence spectra. The theoretical spectrum was obtained from the Evrogen website https://evrogen.comand was corrected with the spectrum of the 561 nm LP filter from Semrock (see Methods section "In vivo imaging and Dendra2 conversion"). The autofluorescence spectrum was from an unconverted Dendra2 mouse with 561 nm excitation. Non-negative least squares regression was used in the linear unmixing algorithm to obtain the intensity of the rD2 fluorescence spectrum and the relevant autofluorescence spectrum. To quantify the amount of rD2 over time, the rD2 intensity was normalized to the autofluorescence intensity to control for variations in laser intensity and optical alignment of the mouse eye.

## Results

### Dendra2-expressing retinal microglia can be efficiently photoconverted in vivo

**Fig. 1:**
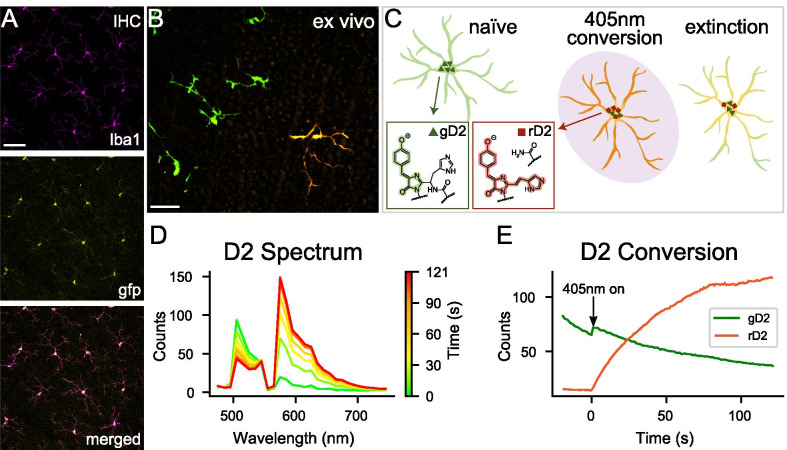
Characterization of the *Cx3cr1*–*Dendra2* mouse. **A** Immunohistochemistry of *Cx3cr1*^*D2/D2*^ retina showing Iba1^+^ microglia staining (top) is concurrent with Cx3cr1–Dendra2 expression (amplified using anti-GFP antibody, middle) and displays normal microglia morphology. Scale 500 µm; from a female mouse, *n* = 3 retina (3 mice). **B** Merged image of live retinal explants from a male *Cx3cr1*^*D2/D2*^ mouse showing microglia with intrinsic unconverted gD2 fluorescence under 488 nm light (left) and a single photoconverted cell expressing rD2 under 560 nm light (right). Scale 100 µm, *n* = 6 retinas (5 mice). **C** Schematic of photoconversion. Naïve microglia shown in green; triangles indicate gD2. Following noninvasive 405 nm light exposure (center), gD2 permanently converts to rD2 (red squares), shifting microglia fluorescence to a red/orange color. Over time, protein turnover causes an extinction of rD2 and gain of gD2 (right). Insets show gD2 and rD2 structure. **D** D2 spectral analysis of the photoconverted cell in **B** shows that prior to photoconversion, the combined emission spectrum from 488 and 560 nm excitation has a strong gD2 primary peak at 505 nm and a slight rD2 secondary peak at 571 nm (green trace, time 0 s). Exposure to 2 min of 405 nm light decreases the gD2 peak and sharply increases the rD2 peak (red trace), reflecting rapid photoconversion (time course shown in multicolored traces). **E** D2 conversion rates of the photoconverted cell in **B** indicate that the 488 nm laser caused gD2 photobleaching prior to 405 nm onset but did not cause appreciable photoconversion (green trace). Onset of the 405 nm laser at *t* = 0 caused a rapid rise in rD2 (red trace) and a decrease in gD2

*Cx3cr1*^*D2/D2*^ mice were generated using CRIPSR/Cas9 to knock-in the Dendra2 (D2) coding sequence behind the Cx3cr1 promoter at the same location previously used to generate *Cx3cr1*^*GFP/GFP*^ mice [25]. Immunohistochemical staining of fixed retinas of *Cx3cr1*^*D2/D2*^ mice showed that D2 was expressed in all Iba1^+^ retinal cells, the vast majority of which are microglia (Fig. 1A). These microglia appeared at normal density with ramified morphologies, suggesting the expression of the exogenous protein had no ill effect on microglia viability or immune activation status. No other retinal cells showed expression of D2.

In live retinal explants of *Cx3cr1*^*D2/D2*^ mice, naïve microglia fluoresced green and had a red–orange color immediately after photoconversion with 405 nm light (Fig. 1B, C, Additional file 1: Movie S1). Spectral analysis of the emitted light allowed quantitative measurement of unconverted green Dendra2 (gD2) and photoconverted red Dendra2 (rD2) over time. Prior to photoconversion, the combined emission spectrum from 488 and 560 nm excitation showed a strong primary peak at 505 nm corresponding to gD2 and a small secondary peak at 571 nm corresponding to rD2 (Fig. 1D, green trace at time 0 s). During the 488 nm scanning, the 505 nm signal noticeably decreased, while the red 571 nm signal did not change, consistent with photobleaching of gD2 that did not cause photoconversion to rD2 (Fig. 1E, green trace before 405 nm onset). Following 405 nm laser exposure for 2 min (19 µW; 2175 J/cm^2^ radiant exposure), the primary green peak decreased, and the secondary red peak increased, reflecting rapid photoconversion from gD2 to rD2 (Fig. 1D; yellow, orange, and red traces). Interestingly, the rate of increase of rD2 fluorescence was much greater than the rate of decrease of gD2 fluorescence (Fig. 1E, compare orange and green traces), as has also been noted by other groups [14, 17, 37, 38]. The complete conversion of all D2 protein within a cell never occurred such that some gD2 protein was still detectible after 405 nm excitation in all experiments.

**Fig. 2:**
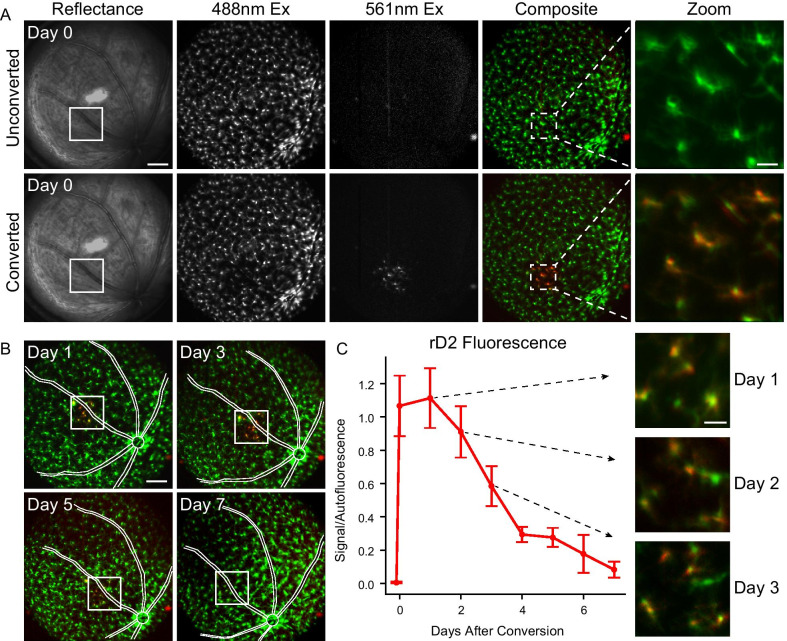
In vivo imaging of Cx3cr1–Dendra2 in healthy retina. **A** Retinal microglia in a *Cx3cr1*^*D2/D2*^ retina from a female mouse appeared qualitatively normal in density and distribution using in vivo SLO under 488 nm excitation both before (unconverted) and immediately after (converted) exposure of a small retinal area to 405 nm light (75 µW, 365 × 365 µm area, 9 J/cm2 radiant exposure). The 405 nm exposure resulted in a clearly defined region of rD2 fluorescence; rD2 was not observed in surrounding regions or in the unconverted eye. Composite image shows a pseudo-colored merge of the 488 nm and 561 nm images; zoomed region denoted by dashed lines. Scale 300 µm; zoom scale 50 µm, n = 8 retinas (5 mice). **B** Changes in rD2 fluorescence over the course of 1 week taken from the same eye as shown in **A**. Converted region denoted with a white box; blood vessels drawn for reference. Scale 300 µm. **C** Quantification of rD2 using spectral analysis taken over 7 days (mean ± SEM, *n* = 5 retinas). Insets show zoomed images taken from the same mouse as in **A** at days 1–3 after photoconversion. Scale 50 µm

We next imaged the retinas of *Cx3cr1*^*D2/D2*^ mice in vivo with combined Scanning Laser Ophthalmoscopy (SLO) to visualize the fluorescent cells and Optical Coherence Tomography (OCT) with three-dimensional light scattering to visualize the retinal architecture. Similar to ex vivo live observations, in vivo SLO imaging with 488 nm excitation revealed a retinal microglial population that appeared qualitatively normal in density and distribution, with no obvious rD2 fluorescence (Fig. 2A, unconverted). After 405 nm exposure, there was a large increase in rD2 fluorescence and a concomitant decrease in gD2 fluorescence in the converted area (Fig. 2A, converted). Photoconverted cells could be tracked in vivo over multiple days, with rD2 fluorescence gradually decreasing over the course of a week (Fig. 2B). Spectroscopy was used to quantify in vivo changes accurately and objectively in a subset of cells across a small area (219 × 219 µm) over time (Fig. 2C). The disappearance of red fluorescence is expected based upon the gradual protein turnover of the rD2 protein, leaving only newly translated gD2 within existing cells; however, at the current resolution of our in vivo imaging, it was not possible to differentiate between loss of the rD2 protein and disappearance of red fluorescent cells themselves.

Of note, a high fluence of 405 nm light (9 J/cm^2^) increased OCT light scattering in the scanned area in 3 out of 4 retinas imaged, denoting light-induced retinal damage; however, lower fluences of 405 nm light (4.5–7 J/cm^2^) did not cause any detectable retinal damage, while still permitting sufficient photoconversion to readily tag individual microglia with our imaging system. In some retinas, low fluences of 405 light (4.5–7 J/cm^2^; 5 min duration) that resulted in photoconversion also resulted in recruitment of microglia to the converted area. This was surprising, since there were no corresponding changes in OCT light scattering that are commonly associated with local retinal damage. In all cases, any recruited microglia returned to their normal mosaic spatial distribution within a week.

**Fig. 3:**
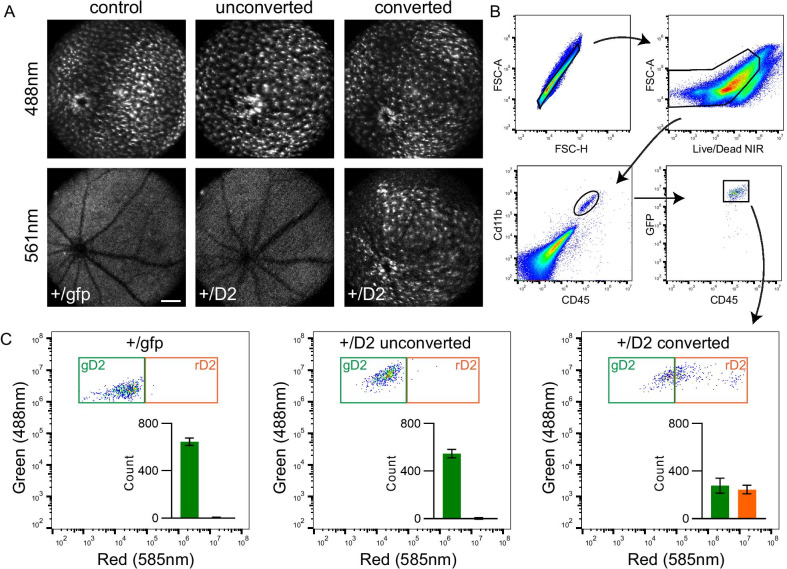
Quantification of photoconverted D2 cells using flow cytometry. **A** In vivo SLO imaging of a *Cx3cr1*^+*/GFP*^ retina, a naive *Cx3cr1*^+*/D2*^ retina (unconverted), and a photoconverted *Cx3cr1*^+*/D2*^ retina that was exposed to 405 nm light across the entire retinal surface (110 µW, 780 × 780 µm per quadrant, 3.5–5 J/cm^2^ radiant exposure). Scale 300 µm. **B** Flowcytometry gates used to identify single cells, alive, double positive for Cd11b^+^ CD45^+^, and with high expression of Cx3cr1, as measured by green fluorescence to exclude peripheral monocytes. **C** Quantification of green gD2 cells and green and red double positive rD2 cells from each condition. The Cx3cr1^+/GFP^ retina was composed entirely of green, GFP^+^ microglia, similar to the Cx3cr1^+/D2^ unconverted retina which was nearly all green gD2 microglia, with a negligible population of rD2 fluorescence. Conversely, the Cx3cr1^+/D2^ photoconverted retina had fewer gD2 cells and a large population of rD2 cells. Error bars are SE, n = 3 retinas per group (5 mice), all retinas were from male mice

To quantify the photoconverted and non-converted microglial populations in vivo, we used fluorescence activated cell sorting (FACS). Since Cx3cr1 has been shown to have immunomodulatory and neuroprotective effects in the retina [12, 39, 40], for this and all subsequent experiments, heterozygous mice (e.g., *Cx3cr1*^+*/D2*^) were used to ensure microglia possessed a functional copy of Cx3cr1 and a single copy of the D2 label. We converted four large areas in one eye of a *Cx3cr1*^+*/D2*^ mouse, creating a large subpopulation of rD2 microglia across the retina (Fig. 3A). Within 24 h after conversion, mice were euthanized and the retinas removed; *Cx3cr1*^+*/GFP*^ retinas were processed in parallel for comparison. Retinas were dissociated and all live, single cells expressing CD45^+^ Cd11b^+^ with high Cx3cr1 expression (as measured by GFP fluorescence) were quantified with flow cytometry, using the gating shown in Fig. 3B. As expected, the *Cx3cr1*^+*/GFP*^ mouse retina contained GFP-expressing cells but no red fluorescence (Fig. 3C, left). The unconverted control eye of the *Cx3cr1*^+*/D2*^ mouse was similar, with only a few rD2 cells detected (Fig. 3C, middle). However, the converted eye in the *Cx3cr1*^+*/D2*^ mouse showed fewer gD2 microglia and a greatly increased number of rD2 microglia (Fig. 3C, right). Interestingly, the green fluorescence was noticeably brighter in the *Cx3cr1*^+*/D2*^ mice compared to the *Cx3cr1*^+*/GFP*^ mice (Fig. 3C). These results demonstrate that expression from a single copy of D2 and its photoconversion in vivo are sufficient to be used for flow cytometry and cell sorting.

### Photoconverted Dendra2 to identify resident microglia during widespread retinal degeneration

**Fig. 4:**
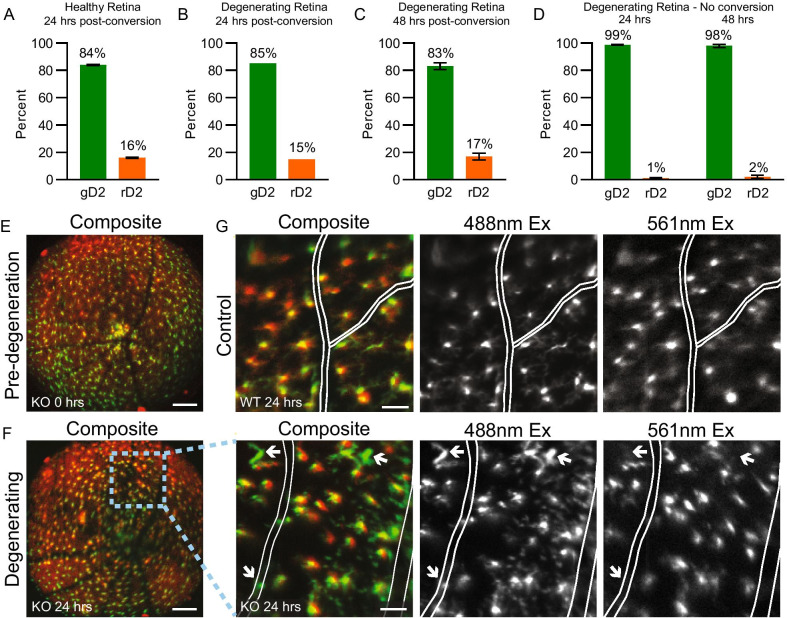
Photoconversion is stable for several days and can differentiate subpopulations of microglia in healthy and degenerating retina. **A** In healthy, dark reared *Arr1*^*−/−*^* Cx3cr1*^+*/D2*^ mice, 24 h after a large portion of retina was photoconverted in vivo (150 µW, 780 × 780 µm, 6 J/cm^2^ radiant exposure), 16% of flow cytometry-isolated cells were rD2 positive. **B**, **C** Similar numbers were found in degenerating *Arr1*^*−/−*^* Cx3cr1*^+*/D2*^ retina after 24 h **B** and 48 h **C** of light exposure, demonstrating stability of the photoconverted microglia population over multiple days in degenerating retina. **D** In degenerating retinas that were not subjected to photoconversion, only 1–2% of cells were rD2 positive, which is similar to unconverted controls in healthy retina. Error bars are SE, n = 2 retinas, 1 male/1 female for each experimental condition. **E**–**G** In *Arr1*^*−/−*^* Cx3cr1*^+*/D2*^ (KO) and *Arr1*^+*/*+^ *Cx3cr1*^+*/D2*^ (WT control) mice, resident microglia in the imaging area were photoconverted (6 J/cm^2^) prior to light exposure. Comparison of pre-degenerating **E** and degenerating **F** retina in the same mouse revealed that after 24 h of light exposure, bright green cells that had not been photoconverted were visible within and near retinal vessels only in degenerating retina (**F**, white arrows in zoomed images) and were clearly distinguishable from resident microglia that appeared yellow/orange and contained gD2 and rD2. Similar bright green, single labeled cells were not observed in *Arr1* wildtypes **G**. Images **E**, **F** from same female mouse, **G** from a male mouse; panels to the right of **F** are digitally magnified images from the dashed box area in the full imaging field to the left; white lines denote vasculature in zoomed images. Widefield scale 300 µm, zoomed scale 100 µm

Having shown that flow cytometry could be performed immediately after in vivo photoconversion, we next used FACS to quantify photoconverted cells over time in a model of neurodegeneration. Mice lacking Arrestin-1 (Arr1, also known as visual arrestin) were used to noninvasively induce photoreceptor degeneration. Arr1 is a photoreceptor-specific cytosolic protein that deactivates photoexcited rhodopsin, shutting down the rod phototransduction cascade [27, 41]. In *Arr1*^*−/−*^ mice, rods become extraordinarily sensitive to light but continue to be healthy as long as the animals are dark-reared; prolonged exposure to room light induces photoreceptor degeneration [28, 42–44], resulting in the loss of half of the photoreceptors within 48 h [8]. *Arr1*^*−/−*^* Cx3cr1*^+*/D2*^ mice were dark-reared and their microglia photoconverted in four large areas in one eye of each mouse, either prior to (“Healthy Retina”, Fig. 4A) or after 4 days of light-induced photoreceptor degeneration (“Degenerating Retina”, Fig. 4B–D). Quantification of gD2 and rD2 cells in the retina by FACS revealed that the population of photoconverted rD2 microglia was remarkably stable regardless of the health status of the retina for up to 48 h (Fig. 4A–C). As before, the unconverted control eyes showed negligible rD2 cells (Fig. 4D). Together, these data demonstrate that subpopulations of retinal microglia can be reliably quantified in a degenerating retina for at least 48 h after photoconversion, and that there is no detectable microglial cell loss during this time of profound photoreceptor clearance.

Next, we used this degeneration model to monitor cell subpopulations during retinal degeneration in vivo using SLO retinal imaging. In healthy mice (prior to inducing degeneration), we photoconverted resident microglia across the full area of the visible retina using the 405 nm laser (Fig. 4E). Both control (*Arr1*^+*/*+^ *Cx3cr1*^+*/D2*^*)* and experimental mice (*Arr1*^*−/−*^* Cx3cr1*^+*/D2*^) were then exposed to continuous room light, initiating retinal degeneration in the *Arr1*^*−/−*^ mouse. Within 24 h, green cells that had not been photoconverted were visible within and near retinal vessels only in the degenerating retinas (Fig. 4F, white arrows). In contrast, retinas of WT mice 24 h after conversion contained only a population of yellow/orange cells and no green cells (Fig. 4G). Since resident microglia contained both gD2 and rD2, giving them a yellow/orange appearance in the composite image, the bright green cells that were easily distinguishable from the rD2-containing population in the degenerating *Arr1*^*−/−*^ retinas likely originated from outside the retinal field of view, presumably having extravasated from the retinal vasculature as previously described [8].

### Microglia with converted Dendra2 can be tracked during a response to localized photoreceptor damage

**Fig. 5:**
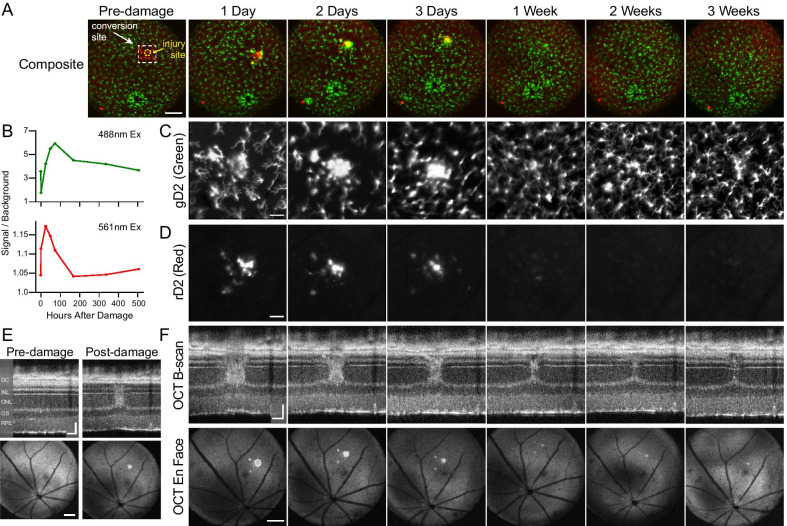
Microglial response to localized damage. **A** Composite widefield images showing naïve microglia (green) and photoconverted microglia (red) in the retina prior to focal NIR damage (pre-damage) and the subsequent 1 day to 3 weeks in the same female mouse (*n* = 1 retina). Cells were photoconverted in an area of ~ 365 × 365 µm (9 J/cm^2^, dashed white line); retinal damage was induced at the injury site (yellow, ~ 150 µm diameter). All detectable rD2 microglia migrated to the injury field within 24 h, progressively forming a dense, bright cluster of cells over the first 3 days. Scale 300 µm. **B** Quantified intensity of gD2 (green, top trace) and rD2 (red, bottom trace) fluorescence in the injury site shown in **A** over time. The gD2 signal was initially bright against the background but lost nearly twofold intensity after photoconversion (lower point at *t* = 0); conversely, the rD2 signal increased nearly threefold after photoconversion (upper point at *t* = 0), peaking 24 h after damage, then falling to baseline levels by 1 week. **C**, **D** Magnified views of the gD2 (488 nm excitation) and rD2 (561 nm excitation) fluorescent images at each timepoint within the injury locus. Scale 100 µm. **E** OCT imaging was performed simultaneously to monitor the integrity of retinal layers and the changes in light scattering characteristic of retinal damage in the same mouse as **A**. Both OCT B-scans (top, optical sections) and en face images (bottom) through the injury locus reveal the increase in light scattering in the outer nuclear layer (ONL) evoked by the NIR laser damage (Post-damage). Note that prior to NIR laser exposure (Pre-damage) retinal layers appeared normal, confirming that the prior 405 nm light exposure used for D2 photoconversion did not cause photoreceptor damage. **F** OCT B-scans (top) and en face images (bottom) for subsequent timepoints matched to corresponding SLO in **A**. The localized damage from the NIR exposure was most extensive at 1 day and nearly resolved by 3 weeks. B-scan scale 100 µm horizontal, 50 µm vertical; en face scale 300 µm. *GC* ganglion cell layer, *INL* inner nuclear layer, *OS* outer segments, *RPE* retinal pigment epithelium

One of the great promises of this mouse line is the ability to track individual microglia over space and time in vivo. Thus, we used local laser damage in the retinas of *Cx3cr1*^+*/D2*^ mice to study how cells move to and from a local site of injury as the damaged cells are removed and the tissue reorganizes. Microglia were first photoconverted in a small retinal region (Fig. 5A, conversion site) without causing damage (Fig. 5E, pre-damage OCT). Subsequently, photoreceptors within the rD2 field were damaged using a high intensity near-infrared (NIR) laser (Fig. 5A, injury site), causing an immediate increase in OCT light scattering indicative of tissue damage (Fig. 5E) that could be monitored over time (Fig. 5F).

After NIR damage, all rD2-labeled microglia migrated to the injury field and progressively formed a dense, bright cluster of cells over 3 days (Fig. 5A, zoom 5D). Initially the rD2 cells accumulated on the side of the injury field closest to the region from which they had migrated. Over a period of days, the rD2 cells became dispersed throughout the injury field. After 1 week, the structural damage visible by OCT was less distinct and most of the microglia had left the injury locus; only a small cluster of fluorescent cells occupied the core damaged region (Fig. 5A, zooms 5C, D). By this time, the rD2 label had become difficult to detect, with only a few faint flocculent red-fluorescent bodies visible (Fig. 5D, 1 week timepoint). Finally, at 2 and 3 weeks after NIR damage, no rD2 label was at all detectable and the green microglia distribution had largely returned to normal, with only a small cluster of gD2 cells remaining in the core of the injury field (Fig. 5A, zooms 5C, D).

To track the microglial response and the change in rD2 fluorescence over time, we quantified the intensity of gD2 and rD2 fluorescence from the SLO images, following an established protocol [33]. The SLO gD2 signal was initially prominent but lost nearly twofold intensity after 405 nm conversion (Fig. 5B, 488 nm Ex). This gD2 signal subsequently increased over time, peaking at 72 h presumably due to the accumulation of cells at the injury locus. From 1 to 3 week post-damage, the gD2 signal decreased, returning nearly to baseline by 3 weeks. Image analysis of the emitted light showed an immediate loss of gD2 and gain of rD2 signal after 405 nm exposure, consistent with photoconversion (Fig. 5B, 561 nm Ex). After 24 h, the rD2 signal began to gradually fall, presumably as protein levels in the cell turned over, reaching baseline around 7 days (Fig. 5B). Overall, these results show that distinct microglial populations can be reliably tracked over space and time as they respond to neuronal injury and degeneration.

## Discussion

Here we show that the *Cx3cr1*–*Dendra2* mouse (*Cx3cr1*^+*/D2*^) is a useful model for labeling resident microglia and unambiguously tracking immune subpopulations for several days during neuronal injury and tissue remodeling.

In the *Cx3cr1*^*D2/D2*^ homozygous mouse, the endogenous Cx3cr1 alleles have been replaced by knock-ins of the fluorescent protein Dendra2, the same strategy employed in the commonly used *Cx3cr1*^*GFP/GFP*^ mouse [25, Jackson Labs strain 005582]. Healthy neurons express the receptor’s ligand, Cx3CL1, which helps maintain microglia in a normal basal state, and studies indicate that mice heterozygous for *Cx3cr1*^+*/GFP*^ have microglia that function normally in the retina [40, 45, 46]. For our study, we likewise used heterozygous *Cx3cr1*^+*/D2*^ mice and did not observe any qualitative abnormalities in microglia form or function. Histology and in vivo SLO imaging of retinal microglia showed a ramified morphology and spatial distribution that is typical for cells in healthy CNS retina. Following photoreceptor damage, these microglia underwent a morphological change and migrated to the affected photoreceptors, a functional response indistinguishable from observations in wild-type (C57BL/6 J) and *Cx3cr1*^+*/GFP*^ mice [1, reviewed in 12, 47]. Together, these data indicate that microglia in the *Cx3cr1*^+*/D2*^ mouse retain their ability to respond to neurodegeneration and function comparably to those in the *Cx3cr1*–*GFP* mouse.

While naïve *Cx3cr1*^+*/D2*^ cells continually produce green GFP-like fluorescence, photoconversion in cells of interest can be efficiently driven by noninvasive 405 nm light exposure, converting selected cells to a permanent RFP-like fluorescence that lasts up to a week (Figs. 1, 2, 5). Once photoconverted, rD2 can be tracked both ex vivo and in vivo in healthy and degenerating retinas, and the D2 protein is stable enough to survive tissue processing for flow cytometry (Figs. 3, 4). In principle, the *Cx3cr1*^+*/D2*^ mouse should also be useful for studying rates of protein turnover, though we did not use it for this purpose. In our in vivo studies that imaged the same cell populations across several days, cell crowding and intermixing did not allow quantitative interpretation of the rD2 and gD2 ratio to estimate protein turnover rates. Overall, the ability to track, sort, and quantify naïve gD2 and photoconverted rD2 microglia simultaneously is a powerful tool for disentangling the roles of mixed immune cell populations.

A variety of other tools have been developed to fluorescently label resident microglia and infiltrating macrophages and visualize their dynamics during health and disease [25, 48–53]; however, very few of these are able to target and track spatially distinct subsets of microglia that are known to exist [4–7]. One exception is the *Cx3cr1*–*CreER* mouse that has been used to develop the “Microfetti” mouse in which individual long-lived macrophages are labeled with one of four fluorophores [54]. This mouse allows subsets of microglia to be distinctly labeled and has been useful for better understanding microglia proliferation at rest and during activation. However, in this line the fluorophore expression after tamoxifen administration is random, and thus spatially distinct subsets of microglia cannot be purposefully targeted. In contrast, the *Cx3cr1*^+*/D2*^ mouse described here allows individual, spatially distinct subsets of microglia, at varying scales, to be specifically tagged and followed over time using a variety of in vivo*, *ex vivo*,* and histological techniques.

In this study, we utilized the *Cx3cr1*–*Dendra2* mouse to noninvasively tag and track subpopulations of cells in the retina. Specifically, in response to widespread degeneration, photoconverted rD2 resident microglia were joined by naïve gD2 circulating Dendra2-expressing cells that exited the retinal vasculature and infiltrated the parenchyma (Fig. 4E, white arrows). Furthermore, following local laser damage, a subpopulation of photoconverted microglia clustered at the injury locus (Fig. 5, rD2 at 1–3 days) then redistributed as the tissue recovered (Fig. 5B, 1–3 weeks). This ability to specifically label subsets of cells could also permit functional comparisons between distinct populations of macrophages using a variety of downstream techniques that typically do not include spatial information, such as single-cell RNAseq and ATAC-seq. Thus, this mouse can be used both to track distinct macrophages over time in the tissue and to probe specific roles of unique immune populations during injury and degeneration.

## Conclusion

Distinguishing between subpopulations of microglia and other immune cells within the CNS during episodes of neuronal death and tissue remodeling has proven to be challenging. Here we describe the novel *Cx3cr1*–*Dendra2* mouse (*Cx3cr1*^+*/D2*^) in which subpopulations of microglia can be photoconverted from green to red and tracked unambiguously for several days during neuronal injury and tissue remodeling. Naïve and photoconverted Dendra2 signal can also be used for flow cytometry and cell sorting of distinct microglia populations. This mouse line has additional uses beyond retinal microglia studies, such as tracking resident immune cells in other CNS compartments, quantifying protein turnover within microglia, and studying the function of Cx3cr1-expressing myeloid cells outside the CNS.

## Supplementary Information

**Additional file 1: Multimedia.** Movie S1—Zoomed image of a ex vivo microglia during 405 nm laser photoconversion. There is an immediate decrease in 488 nm green fluorescence and an increase in 561 nm red fluorescence, which increased upon each subsequent scan of the 405 nm laser. Images from a male *Cx3cr1*^*D2/D2*^ mouse.

## Data Availability

The data sets created during the current study are available from the corresponding author on reasonable request.

## Abbreviations

Arr1: Arrestin-1
CNS: Central nervous system
D2: Dendra2
DMEM: Dulbecco's modified eagle medium
FACS: Fluorescence activated cell sorting
FBS: Fetal bovine serum
GC: Ganglion cell layer
gD2: Green fluorescent Dendra2 protein
GFP: Green fluorescent protein
INL: Inner nuclear layer
KO: Knock-out
LUT: Look-up table
NIR: Near infrared
OCT: Optical coherence tomography
OS: Outer segments
PBS: Phosphate buffered saline
PBT: Phosphate buffered saline, bovine serum albumin, and triton x-100
rD2: Red fluorescent Dendra2 protein
RFP: Red fluorescent protein
ROI: Region of interest
RPE: Retinal pigment epithelium
SE: Standard error
SLO: Scanning laser ophthalmoscopy
WT: Wild type
